# Factors associated with the high prevalence of oesophageal cancer in Western Kenya: a review

**DOI:** 10.1186/s13027-017-0169-y

**Published:** 2017-11-03

**Authors:** Gabriel Kigen, Naftali Busakhala, Zipporah Kamuren, Hillary Rono, Wilfred Kimalat, Evangeline Njiru

**Affiliations:** 1 Department of Pharmacology & Toxicology; Department of Hematology & Oncology, Moi University School of Medicine, P.O. Box 4606-30100, Eldoret, Kenya; 2 Department of Pharmacology & Toxicology; Department of Hematology & Oncology, Moi University School of Medicine, P. O. Box 4606-30100, Eldoret, Kenya; 3 Department of Pharmacology & Toxicology, Moi University School of Medicine, P.O. Box 4606-30100, Eldoret, Kenya; 4 Kitale County Hospital; London School of Tropical Medicine & Hygiene, P.O. Box 98-30200, Kitale, Kenya; 5 Retired Permanent Secretary, Ministry of Education, Science & Technology, Provisional Administration & Internal Security, Office of the President, P. O. Box 28467-00200, Nairobi, Kenya; 6 Department of Internal Medicine; Department of Hematology and Oncology, Moi University School of Medicine, P.O. Box 4606, Eldoret, 30100 Kenya

**Keywords:** Oesophageal carcinoma, High prevalence, Causes, Mycotoxins, Kalenjin

## Abstract

Oesophageal carcinoma (OC) is highly prevalent in Western Kenya especially among the members of the Kalenjin community who reside in the Northern and Southern areas of the Rift Valley. Previous authors have suggested potential association of environmental and genetic risk factors with this high prevalence. The environmental factors that have been suggested include contamination of food by mycotoxins and/or pesticides, consumption of traditional alcohol (locally referred to *“Busaa”* and *“Chan’gaa”*), use of fermented milk *(“Mursik”)*, poor diet, tobacco use and genetic predisposition. The aim of this paper is to critically examine the potential contribution of each of the factors that have been postulated to be associated with the high prevalence of the disease in order to establish the most likely cause. We have done this by analyzing the trends, characteristics and behaviours that are specifically unique in the region, and corroborated this with the available literature.

From our findings, the most plausible cause of the high incidence of OC among the Kalenjin community is mycotoxins, particularly fumonisins from the food chain resulting from poor handling of cereals; particularly maize combined with traditional alcohol laced with the toxins interacting synergistically with other high-risk factors such as dietary deficiencies associated alcoholism and viral infections, especially HPV. Urgent mitigating strategies should be developed in order to minimize the levels of mycotoxins in the food chain.

## Background

Oesophageal carcinoma (OC) is the sixth cause of cancer related mortality, and the eighth most common cancer globally. It is more common among men, and associated with older age [1–3]. There are two histological types; oesophageal adenocarcinoma (OAC) which presents in the lower part of the oesophagus, and oesophageal squamous-cell carcinoma (OSCC) which tends to be on the upper and middle part, and arises from epithelial cells that line the oesophagus [4–8]. Several predisposing risk factors for OC have been reported [9–11]. The risk factors that have been reported for OSCC include tobacco smoking and consumption of alcohol [12–14], consumption of hot food and beverages [15–18], including *“Mate”,* a tea-like nonalcoholic infusion from *Ilex paraguariensis* plant [19, 20] and poor diet [10]. The main risk factors that have been reported to predispose OAC include gastroesophageal reflux disease, obesity, smoking, aging, diet and radiation therapy [5, 14, 21–24]. Factors known to protect against adenocarcinoma development include *Helicobacter pylori* infection, a diet rich in fruits and vegetables, and use of aspirin and other non-steroidal anti-inflammatory agents [10, 25, 26]. The role of human papilloma virus (HPV), an oncogenic virus is still debatable [27]. Some researchers have concluded that it may have a role in the OSCC carcinogenesis based on meta-analytic reviews [28–30], while others have concluded that it has no direct role [31–33]. Authors from a recent publication have also provided evidence of the lack of HPV association based on molecular biology criteria [34]. In addition, recent genomic studies obtained from sequencing OSCC samples collected from sub-Saharan Africa (SSA), suggest that the endemic nature of the disease is due exposure to a carcinogen other than tobacco and oncogenic viruses [35].

The incidences vary globally, with OAC being more prevalent in developed world; and OSCC in low and middle income countries (LMICs) with Eastern and Southern Africa, as well as Eastern Asia recording the highest prevalence [1, 8, 14, 36–38]. It has also been reported to be more prevalent in black men [39–41]. This has largely been attributed to poor diet/dietary deficiencies, alcohol consumption and tobacco smoking, exposure to mycotoxin metabolites (aflatoxins and fumonisins in particular) and HPV infection, especially in SSA [37, 42–45]. Interestingly, authors from a fairly recent research paper have reported the age-standardized incidence rates of OSCC in East Africa to be higher than the mean world rates, suggesting that the incidence in the region may be higher than initially postulated [46]. Currently, it is difficult to get accurate national data on the prevalence of OC in Kenya because there only two cancer registries, one based in the capital Nairobi (Nairobi Cancer Registry [NCR]) and the other in Eldoret (Eldoret Cancer Registry) which is located in the Western region respectively [47, 48]. Data from NCR have reported OC to be the second most diagnosed cancer in men (8.9%) after prostate, and third in women (4.9%), after breast and cervical [49]. A new report from NCR classifying cancer risk as per ethnic group still has OC as the third most frequent [50].

However, OSCC has been reported to occur more frequently in Western Kenya, and more significantly among the members of the Kalenjin community who reside in the Northern and Southern areas of the Rift Valley [51–58]. Five-year data from Eldoret Cancer Registry regarding cancer prevalence in Uasin Gishu County, an area resided mainly by the Kalenjins shows OC to be the most common cancer. It is most prevalent among men followed by leukemia and prostate, whereas it is third most common in women after cervix and breast [48]. It has been reported to literally occur in all the age groups, with OSCC situated at the middle third portion of the oesophagus being the commonest histological type [53, 54, 56, 57, 59]. Previous authors have suggested potential association of environmental and genetic risk factors with this high prevalence. The environmental factors that have been suggested include consumption of traditional alcohol (locally referred to *“Busaa”* and *“Chan’gaa”*), use of fermented milk *(“Mursik”)*, poor diet and exposure to mycotoxins and nitrosamines [37, 54, 57, 60–62].

The North and Southern parts of Rift Valley comprise an important agricultural area in Kenya. Indeed, it has been dubbed at the “bread basket” of the country. The Kalenjin constitute the largest community residing in the region, with Kalenjin sub-tribes of Nandi, Keiyo and Marakwet living in the northern part; while the Kipsigis and their Maasai cousins residing in the southern part. The Luhya community is mainly found in the neighbouring region (formerly referred to as Western Province), and some parts of the North rift. Naturally, the main activity in the region is farming, in both small and large scales. A type of stiff porridge made by mixing cornmeal with boiling water, commonly referred to as *“Ugali”* is the most popular dish in the area; and is mainly consumed with vegetables, and occasionally meat for those who can afford [63, 64]. This is usually combined with milk, either fresh or fermented (*“Mursik”*) among the Kalenjin [65]. Alcoholism is rampant especially in the rural areas, with the most popular drink being traditional brews, either *“Busaa”* or *“Chan’gaa”* [66, 67]. Incidentally, this is the region that unusually bears one of the highest brunts of OC in the world, especially among the Kalenjin community [46, 48, 52–54, 57, 58]. We have critically examined the potential contribution of each of the factors that have been postulated to be associated with the high prevalence of the disease in order to establish the most likely cause. We did this by analyzing the trends, characteristics and behaviours that are specifically unique in the region, and corroborated this with the available literature. These include the roles of contamination of food by mycotoxins and/or pesticides, consumption of traditional alcoholic brews and *“Mursik”*, tobacco use, genetic predisposition and HPV infection.

### Mycotoxins

Mycotoxins are toxic metabolites produced by fungi that normally contaminate agricultural cereals, either in the field, during harvest or storage; and are mainly associated with cereal crops, including maize, wheat, barley, rice and oats. They are common throughout the world, and mycotoxin contamination of food is now considered a serious public health problem, especially in SSA [68, 69]. They include aflatoxins, fumonisins, zearalenone, moniliformin, ochratoxins, trichothecenes, deoxynivalenol, diacetoxyscirpenol, and nivalenol [43, 70, 71]. *Aspergillus flavus* and *Aspergillus parasiticus* which are abundant in warm and humid regions are the main aflatoxin producing group of fungi, whereas fumonisins and trichothecenes are mainly produced by *Fusarium verticillioides* and *F. proliferatum* [45, 72–74]. Mycotoxins metabolites have been associated with the development of several diseases including aflatoxicosis, hepatotoxicity, neural tube defects, immunosuppression, infertility, haematotoxicity, growth impairment and cancer [74–78]. The metabolites that have been described to induce oncogenesis include fumonisins and aflatoxins, which have both been associated with the development of oesophageal and hepatocellular carcinoma [HCC] [79]. Most of the current publications however tend to associate aflatoxins more with HCC, whereas fumonisins are linked to the development of both malignancies [80–83]. Additionally, fumonisins have been associated with renal carcinoma [84, 85].

#### Fumonisins

Fumonisin B_1_ (FB_1_) is the most prevalent member of fumonisin family of toxins, and its link to the development of OC has been known for a long time; having been implicated in the high incidence of OC in China and South Africa in the 90s [76, 81, 86–88]. It is also a known hepatocarcinogen that causes HCC [82, 83, 87–89]. The mechanisms for carcinogenesis are still uncertain. It is however thought to be nongenotoxic (non-DNA reactive), and several mechanisms have been postulated for its oncogenesis. These include possible role of oxidative damage during initiation and disruption of lipid metabolism, integrity of cellular membranes and altered growth-regulatory responses [78, 83, 88]. FB_1_ has been demonstrated to be nongenotoxic in bacterial mutagenesis screens or unscheduled DNA-synthesis assays [84, 90]. Previous researchers have concluded that it produces oncogenesis through apoptotic necrosis, atrophy, and consequent regeneration as a result of ceramide synthase inhibition and disruption of sphingolipid metabolism [82–84, 90–92]. FB_1_ bears a structural similarity to the cellular sphingolipids, thereby disturbing the metabolism of sphingolipids by inhibiting ceramide synthase enzyme leading to accumulation of sphinganine in some cells and tissues thus inducing apoptosis, especially in the liver cells. The ability of FB_1_ to accumulate sphingosine or sphinganine and arrest the cell cycle in selected cells is thought to play an important role in the carcinogenesis or disease [78, 88, 93].

#### Aflatoxins

Aflatoxins are known human carcinogens that have been demonstrated to participate in the pathogenesis of HCC, with aflatoxin B_1_ (AFB_1_) being the most dominant and potent aflatoxinin [80, 81, 94, 95]. AFB_1_ in conjunction with hepatitis B virus has been associated with the development of HCC in resource limited countries [72, 85, 96–100]. In addition, the toxic effects of aflatoxins on immunity and nutrition combine to negatively affect health factors including HIV infection which accounts for a high percentage of the burden of disease in developing countries [80, 94]. Indeed, the dietary exposure to aflatoxins has been touted as an important contributor to the high incidence of HCC in Asia and SSA, where almost 82% of the worldwide cases occur [97, 101–103]. The exact mechanism is not yet very clear, but it is thought to be through conversion of aflatoxin B_1_ to aflatoxin B_1_ formaminopyrimidine, a mutagenic and carcinogenic adduct metabolite that acts synergistically hepatitis B virus to generate mutations of the tumour suppressor genes thus resulting in hepatocellular carcinoma [104–108]. Additionally, aflatoxin contamination has also been described as risk factor for esophageal cancer [109].

#### Mycotoxin contamination

There are many published reports about mycotoxin contamination, and its toxic metabolites in Kenyan cereals, particularly maize [60, 61, 110–117]; including outbreaks [118–120]. This has largely been associated with delayed and poor harvesting methods, drying and storage in deficient or inappropriate facilities [115, 116, 119, 121, 122]. Mycotoxins have also been detected in food including meat, milk and eggs; as well as in traditional beer. This has been attributed to contaminated livestock feeds and maize flour used in the fermentation of *“Busaa”* [123, 124].

Taking Uasin Gishu County in the North Rift as a case in point, the rise in mycotoxin contamination, can be historically traced to failed agricultural policies and diminishing Government support over the years [125, 126]. The Kalenjin are pastoral by nature, but during the colonial period (up to late1950s), they like every other indigenous Africans were confined to tribal homelands commonly referred to as “reserves”, where they practiced peasant farming. They grew finger millet and maize (*“Cheborosinik”*) introduced earlier by the British & Portuguese, that was just enough for subsistence, and supplemented with dairy products [63, 65, 127, 128]. The maize was well dried and stored in small barns, thereby exposing the cereal to very low levels of contamination [129–132]. After Independence (1961-1976), land was distributed and the farmers adopted higher yielding varieties of maize and wheat which led to a significant increase in agricultural production. The cereals were marketed by a farmer’s organization named the Kenya Farmers Association (KFA) under strict regulations by both the Ministry of Agriculture and Maize and Produce Board [125, 126]. The farmers were also cushioned by a seasonal credit insurance system (Guaranteed Minimum Returns), in case of any crop failures mainly due changing weather patterns. There was therefore little contamination, or the contaminated products were destroyed before reaching the market [127, 133, 134].

**Fig. 1:**
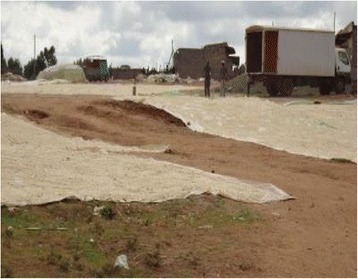
Drying maize in the open

Since the introduction of liberalization of maize produce in 1987/88, farmers have had to endure with a lot of challenges including deregulation of prices, low producer prices, low quality inputs, weather variability, elimination of subsidies, disincentives for production, including inefficiencies in marketing, reduction in the acreage and several other problems [122, 126, 134, 135]. In addition, there has been significant importation of cheap maize over the years, thus dampening the local prices. The ever-changing weather pattern and lack of storage facilities coupled with the high cost of drying has exacerbated the problem. This has led to poverty and poor management of the crop, with farmers adopting poor methods of handling the produce in order to decrease the costs of production. The farmers now tend to dry their maize in open spaces in urban areas when rejected by the millers due to high moisture content, whereby they may not salvage in an event of a sudden downpour, thus exposing the grain to further contamination [136] **(**Fig. 1 **)**. In addition, the rotten pieces are no longer discarded but are either converted to animal feeds or milled and sold to *“Busaa*” brewers [123, 125, 137–139]. This directly introduces mycotoxins to the food chain, either through consumption of *“Ugali”* and milk (both staple diets among the Kalenjin), meat, eggs and other animal products as well as *“Busaa”* [60, 123, 124]. This is evinced by the reports from the region of unacceptably high levels of mycotoxins and its metabolites in stored maize [61, 110–112], and more disturbingly, including milled maize samples [60]. In some instances, the extent of contamination is so high that sorting the harvested maize to remove those with moulds did not reduce the mycotoxin content [114]. High levels of aflatoxin contamination have even been detected in human serum, and this has been correlated with the level of poverty [140, 141]. With regards to *“Busaa”,* a very popular traditional drink in the region [66], it is apparent that the content of mycotoxin and its metabolites would be high in most samples, based on the rampant use of flour from rotten maize for fermentation. Studies conducted in the region have reported as such [124, 139]. However, related studies have reported that commercial beer have very low mycotoxin toxicity confirming the fact that the farmers tend to supply millers with clean maize because of stringent requirements by the millers, but select the rotten pieces to be sold to *“Busaa”* brewers who are not regulated by authorities [137, 142]. We hypothesize that mycotoxin (especially fumonisin type) contamination of food and alcohol is one of the most likely causes of the high prevalence rates of oesophageal, hepatic and related cancers in Western Kenya.

### Alcohol consumption and tobacco smoking

The association between consumption of alcohol and tobacco smoking with the development of OC, especially OSCC has been known for a long time [13, 23, 143–145]. Indeed, they have both been listed as major contributors of OSCC in SSA [42]. They are thought to interact synergistically in the development of OC, and some researchers have even reported a 35-fold increase in the risk resulting combined use of alcohol, tobacco and marijuana [146–149]; and up to 50-fold in heavy smokers and drinkers compared to those who do not drink or smoke [13].

The pathophysiology of alcohol in OSCC is believed to involve acetaldehyde, a toxic ethanol metabolite which is a recognized carcinogen. In addition, ethanol itself stimulates carcinogenesis by inhibiting DNA methylation and by interacting with retinoid metabolism [150–152]. Polymorphisms involving alcohol and aldehyde dehydrogenase, the enzymes that metabolize ethanol have therefore been associated with increased risk. The genotypes that have been identified from several studies are alcohol dehydrogenase-1B (encoded by the *ADH1B* gene) and aldehyde dehydrogenase-2 (encoded by *ALDH2*) respectively. Carriers of these polymorphic genes are discouraged from taking alcohol based on studies conducted in Asia [153–158]. To date there is scant information from the literature about related genetic studies that have been conducted on Africans; despite the reportedly higher prevalence on OSCC among African-Americans, especially those who use alcohol and tobacco [39, 40]. Combination of tobacco and alcohol consumption further increases the risk of OSCC [146, 147, 159, 160]. The molecular mechanisms by which tobacco induces cancer is postulated to be mediated through the exposure of tobacco-specific carcinogenic nitrosamines; 4-(methylnitrosamino)-1-(3-pyridyl)-1-butanone (NNK) and N'-nitrosonornicotine [NNN] [161]. NNK and NNN initiate tumours through a two-fold synergistic process; deleterious mutations in oncogenes and tumor suppression genes resulting in DNA adducts, as well as enhancement of the tumour growth. The nitrosamines promote tumour growth by binding to the nicotinic acetylcholine receptors and stimulating the deregulation of cell proliferation, survival, migration and invasion which provides an enabling environment for the proliferation [161–164].

Members of the Kalenjin community are not heavy tobacco smokers, but their rampant consumption of traditional alcohol *(“Busaa” and “Chan’gaa”)* laced with mycotoxins could be a potential trigger of OSCC.

### Traditional fermented milk *(“Mursik”)*

Fermented milk is part and parcel of Kalenjin culture, and is usually drunk after every meal. Some have even hypothesized that their world-class athletic prowess is associated with the use of *“Mursik”* [165]. Its preparation process involves the use of a gourd *(“Sotet”),* a bow shaped stick *(“Sosiot”)* usually from palm trees and charcoal from selected trees and shrubs *(“Suteiywo”),* the commonest being *Senna didymobotrya*, *Juniperus procera, Plectranthus barbatus, Olea europaea* and wattle trees. The sticks are used to grind embers of the charcoal by pressing against the walls of a gourd in a methodical, circular in and out movement of the hand until the inside of the gourd is evenly covered with fine dust. Boiled milk is then poured into the gourd and allowed to ferment in cool dry conditions. If a new gourd is used, then it has to be first “sweetened” in order remove the bitter taste. This is done by the use of fresh bark from either *Ozoroa insignis, Pappea capensis* or *Ficus thonningii.* The bark is placed inside the gourd which is then filled with water and left to cure for three days [166].

The association of fermented milk including *“Mursik”* with oncogenesis is inconclusive [167–172]. From the literature, the publications that link this mainly relate to presence of acetaldehyde, a carcinogenic substance in sour milk and other contaminants as a result of poor processing [62, 173–175]. Indeed, most of the publications correlating the prevalence in OSCC among the Kalenjin community to the consumption of *“Mursik”* have postulated as such, but there is no direct relationship from literature [54, 58, 62]. In any case, the amount of acetaldehyde from fermented milk may not be as high as the amount from alcohol per se. The most plausible association between *“Mursik”* with OSCC would therefore emanate from *“Mursik”* containing mycotoxins originating from contaminated animal feeds; which would act synergistically with acetaldehyde present in the fermented milk [60, 70, 123, 124, 138, 176]. Further research should be conducted to determine the levels of mycotoxins and aldehydes in *“Mursik”.*

### Pesticides and herbicides

Being an agricultural region, there is widespread use of pesticides and herbicides in the Rift Valley. From the literature, there are several publications linking the use of these chemicals with potential development of several types of cancers [177–179]. Most of these reports however are based on anecdotal evidence and are largely inconclusive, often requiring more studies for definitive conclusions [180–186]. Nevertheless, most of the research that has been conducted to date have reported the absence of association between pesticide use and OC [187–189]. Of note however, is a report by some researchers about a statistical correlation between pesticide use among agricultural workers and development of some forms of cancer later on in life, including OSCC [190].

### Genetic predisposition

To date, there is no specific gene that has been specifically identified whose overexpression will lead to development of OC, although many genes and miRNAs including p53, VEGF, cyclin D1, and miR-21 are considered useful when predicting the prognosis of EC [191–194]. The predisposition to OSCC is mainly associated with life style risk factors including smoking and alcohol use, and specifically on the polymorphisms in the enzymes involved in metabolism of alcohol acting synergistically with nitrosamines from exposure to tobacco [146, 150, 155, 195]. Genetic polymorphisms of the genes encoding alcohol dehydrogenase-1B *(ADH1B)*, aldehyde dehydrogenase-2 *(ALDH2)* and cytochrome P4502E1 *(CYP2E1)* are known to affect the metabolism of alcohol among the Asian populations [153, 154, 158, 196–198]. Indeed, polymorphism in *ADH1B* and *ALDH2* in combination with tobacco by-products have been demonstrated to increase the risk of OSCC by up to 190 times in Japanese population, and predisposition to OSCC [160]. Carriers of these polymorphic genes are therefore discouraged from taking alcohol [10, 156, 159]. Unfortunately, there is scant information from the literature about studies on the impact of these polymorphisms among the Africans despite the reportedly, unusually high incidents of OSCC among the African-American users of alcohol compared to other races [39, 40].

### Human Papillomavirus Infection

HPV infection has for a long time been postulated to be a potential causative agent in the etiology of OC [199]. Despite the fact the hypothesis regarding the link has been controversial and inconclusive [32, 33, 200, 201], there are several reports to date associating the etiology of OC with HPV virus [27, 202–206], especially in high incidence geographical regions [30, 207–211]. The mechanism of oncogenesis has is thought to be through the inactivation of tumour suppressor genes. HPV, particularly 16 and 18 subtypes has been described to interact with p53 and Rb tumour suppressor proteins leading to their loss of function. In addition, HPV-16 E6 has been demonstrated to down-regulate miR-125b, thus activating the Wnt/beta-catenin signaling pathway which promotes tumorigenesis [212, 213]. Despite the Kenya being in the HPV high risk geographical region, results from a research conducted from samples collected in MTRH suggest that HPV may not play a role in the pathogenesis of OSCC [214]. However, we cannot completely rule out the role of HPV on the oncogenesis of OSCC in the region.

## Conclusions

The most plausible cause of the high incidence of oesophageal cancer in Western Kenya, especially among the Kalenjin community is mycotoxins, particularly fumonisins from the food chain combined with traditional alcohol laced with the toxins interacting synergistically with other high-risk factors such as dietary deficiencies associated alcoholism. Genetic predisposition and HPV-infection may also contribute. , Further research (including population-based case-control studies) should be undertaken to establish their roles. Attempts should be made by the regulatory authorities to eliminate or minimize the levels of mycotoxins in the food chain. Urgent mitigating strategies should be developed in order to stem the public health problems arising from the toxins. These include public education to create awareness on the health risks caused by mycotoxins, and enforcement of strict agricultural measures in handling the cereals to minimize contamination.

## Abbreviations

AFB_1_: Aflatoxin B_1_
FB_1_: Fumonisin B_1_
HCC: Hepatocellular carcinoma
HIV: Human immunodeficiency virus
HPV: Human papilloma virus
LMICs: Low and middle-income countries
NCR: Nairobi Cancer Registry
OAC: Oesophageal adenocarcinoma
OC: Oesophageal carcinoma
OSCC: Oesophageal squamous cell carcinoma
SSA: Sub-Saharan Africa
